# Five Plant Natural Products Are Potential Type III Secretion System Inhibitors to Effectively Control Soft-Rot Disease Caused by *Dickeya*

**DOI:** 10.3389/fmicb.2022.839025

**Published:** 2022-02-22

**Authors:** Anqun Hu, Ming Hu, Shanshan Chen, Yang Xue, Xu Tan, Jianuan Zhou

**Affiliations:** Guangdong Laboratory for Lingnan Modern Agriculture, Guangdong Province Key Laboratory of Microbial Signals and Disease Control, Integrative Microbiology Research Center, South China Agricultural University, Guangzhou, China

**Keywords:** *Dickeya*, type III secretion system, HrpL, inhibitor, biocontrol

## Abstract

*Dickeya zeae*, a plant soft-rot pathogen, possesses a type III secretion system (T3SS) as one of the major virulence factors, infecting a wide variety of monocotyledonous and dicotyledonous plants and causing serious losses to the production of economic crops. In order to alleviate the problem of pesticide resistance during bacterial disease treatment, compounds targeting at T3SS have been screened using a *hrpA-gfp* bioreporter. After screening by Multifunctional Microplate Reader and determining by flow cytometer, five compounds including salicylic acid (SA), p-hydroxybenzoic acid (PHBA), cinnamyl alcohol (CA), p-coumaric acid (PCA), and hydrocinnamic acid (HA) significantly inhibiting *hrpA* promoter activity without affecting bacterial growth have been screened out. All the five compounds reduced hypersensitive response (HR) on non-host tobacco leaves and downregulated the expression of T3SS, especially the master regulator encoding gene *hrpL*. Inhibition efficacy of the five compounds against soft rot were also evaluated and results confirmed that the above compounds significantly lessened the soft-rot symptoms caused by *Dickeya dadantii* 3937 on potato, *Dickeya fangzhongdai* CL3 on taro, *Dickeya oryzae* EC1 on rice, and *D. zeae* MS2 on banana seedlings. Findings in this study provide potential biocontrol agents for prevention of soft-rot disease caused by *Dickeya* spp.

## Introduction

*Dickeya* species are necrotrophic, Gram-negative plant pathogens that cause severe disease in a wide range of plant hosts. In particular, the diseases caused by different species in *Dickeya* genus on rice, banana, potato, and taro have become a major threat to agricultural production in recent years (Hussain et al., 2008; Hu et al., 2018; Li et al., 2020; Dong et al., 2021; Huang et al., 2021). There are currently 13 species in this genus, including *Dickeya chrysanthemi*, *Dickeya dadantii*, *Dickeya dianthicola*, *Dickeya paradisiaca*, *Dickeya zeae*, *Dickeya solani*, *Dickeya fangzhongdi*, *Dickeya aquatic*, *Dickeya poaceaephila*, *Dickeya lacustris*, *Dickeya undicola*, *Dickeya oryzae*, and *Dickeya parazeae* (Hu et al., 2018; Hugouvieux-Cotte-Pattat et al., 2019; Oulghazi et al., 2019; Wang et al., 2020; Hugouvieux-Cotte-Pattat and Van Gijsegem, 2021), and among them, *D. zeae*, formerly known as *Erwinia chrysanthemi* pv. *zeae*, can infect a wide variety of monocotyledons and dicotyledons (Hussain et al., 2008), causing severe soft rot in crops and ornamental plants worldwide. For the prevention and control of this pathogen and other pathogenic bacteria, the most widely used measure in fields is mainly through agriculturally antibiotic treatment. Antibiotics achieve the purpose of prevention and control by inhibiting the growth of or directly killing the pathogen. Several chemical compounds including copper, anthium dioxide, formaldehyde, chlorine dioxide, 8-hydroxyquinoline, cetalkonium chloride, benzalkonium chloride, gluytaraldehyde, and others were used to control *Erwinias* soft rot disease (Lund and Lyon, 1975; Letal, 1977; Wyatt and Lund, 1981). Previous studies have shown that kasugamycin, Virginiamycin, or fungicides (acetic acid, boric acid, and bleach) can reduce the incidence of soft rot (Czajkowski et al., 2011). In addition, benziothiazolinoe 3% wettable powder (WP), tetramycin 0.3% aqueous solution (AS), and bismerthiazol 20% WP had better control effect on potato soft rot (Liang et al., 2020). However, a long-term usage of antibiotics has led to the increasing drug resistance of strains, and the WHO has listed antibiotic resistance as one of the three most important public health threats of the 21st century (Rasko and Sperandio, 2010; WHO, 2014). In this content, it is very important to find new alternative solutions for bacterial disease control.

The pathogenicity of *D. zeae* mainly depends on different virulence factors, including extracellular polysaccharides (EPS), plant cell wall degrading enzymes (PCWDEs), phytotoxins, flagellin, and secretion systems (Zhou et al., 2015; Hu et al., 2018; Feng et al., 2019). Type III secretion system (T3SS) is highly conserved in Gram-negative bacteria, including animal pathogens such as *Salmonella*, *Pseudomonas aeruginosa*, *Escherichia coli*, *Chlamydia yersinia*, *Shigella fowleri*, etc. (Hueck, 1998; Keyser et al., 2008; Lu et al., 2018), and plant pathogens such as *Erwinia*, *Pseudomonas syringae*, *Pectobacterium*, *Xanthomonas*, *Ralstonia*, and *Dickeya*, etc. (Hueck, 1998; Rojas et al., 2004; Pique et al., 2015). In *Dickeya* spp., T3SS is encoded by *dsp*/*hrp*/*hrc* gene clusters and plays an extremely important role in pathogenicity (Yang et al., 2002; Yap et al., 2005; Zhou et al., 2015). *hrp* gene expression was inhibited in rich medium, but was induced in nutrient deficient medium as well as in plants (Wei et al., 1992). T3SS is mainly used to directly inject Type III secreted effectors (T3SEs) into host cells by forming a syringe-like Type III secreting device, leading to disease resistance of host plants and hypersensitive response (HR) of non-host plants (Boucher et al., 1987; Yang et al., 2002; Buttner, 2012). In *Dickeya* bacteria, the expression of T3SS is regulated by a master regulator, HrpL, whose expression and activity are controlled under several regulatory cascades (Zeng et al., 2012). On one hand, *hrpL* upregulates many *hrp* genes that encode the T3SS structural and functional proteins, such as *hrpA*, *hrpN*, and *dspE*. T3SS pilus, whose formation relies on a large number of HrpA subunits, is required for effector proteins translocating into plant cells and located at the downstream of the T3SS regulatory pathway (Jin and He, 2001; Zeng et al., 2010; Li et al., 2015). In soft rot *Erwinia amylovora*, the expression of *hrpA* is most influenced by HrpL among the HrpL regulon (McNally et al., 2012). On the other hand, the expression of *hrpL* is regulated by the HrpX/HrpY-HrpS-HrpL pathway at the transcriptional level and the GacS-GacA-RsmB-RsmA pathway at the post-transcriptional level (Yang et al., 2008; Zeng et al., 2012; Yuan et al., 2020). In addition to the above regulatory pathways, It is reported that the amount of functional *rsmB* transcripts in *D. dadantii* have been reduced by polynucleotide phosphorylase (PNPase), resulting in decreased *hrpL* mRNA stability (Zeng et al., 2010). Additionally, SlyA, a SlyA/MarR family regulator, regulates *hrp* genes of the HrpL regulon in parallel with HrpL in *D. dadantii*, which positively regulates the expression of *hrpA* and *hrpN*, and negatively regulates the expression of *hrpL* by downregulating *hrpS* and upregulating *rsmA* (Zou et al., 2012). Therefore, T3SS is critical in pathogen-host interaction.

Usage of T3SS inhibitors that do not affect pathogenic bacterial growth but effectively reduce their virulence has become one of the promising alternatives of antibiotic treatment for bacterial disease control (Marshall and Finlay, 2014; Charro and Mota, 2015). For instance, some small molecules that specifically inhibit T3SS synthesis or function have been identified as T3SS inhibitors (Yuan et al., 2020). In the exploration of T3SS inhibitors drugs targeting T3SS in animal pathogens *Yersinia pseudotuberculosis*, *E. coli*, *Salmonella enterica*, *Chlamydia*, and *P. aeruginosa* have been found, which include salicylidene acylhydrazides (Kauppi et al., 2003; Tree et al., 2009; Layton et al., 2010; Ur-Rehman et al., 2012; Anantharajah et al., 2017; Uusitalo et al., 2017), phenoxyacetamides (Aiello et al., 2010; Zhang et al., 2013; Bowlin et al., 2014), *N*-Hydroxybenzimidazoles (Kim et al., 2009; Marsden et al., 2016), caminosides (Linington et al., 2002, 2006), and naringenin (Vikram et al., 2011). The mechanism of the action of these inhibitors mainly includes inhibition of T3SS gene transcription, toxic protein secretion, and T3SS device assembly. Some plant-derived and chemically synthesized compounds have been identified as T3SS inhibitors in plant pathogens. In *D. dadantii* 3937, the expression of T3SS was induced by plant-derived compounds o-coumaric acid and t-cinnamic acid through the *rsmB*-RsmA pathway (Yang et al., 2008). Previous studies have shown that the plant phenolic compounds and derivatives p-coumaric acid (PCA), cinnamyl alcohol (CA),and trans-4-hydroxycinnamohydroxamic acid play a role in the inhibition of T3SS expression of *D. dadantii* 3937 (Li et al., 2009, 2015; Joshi et al., 2021). Plant phenolic compounds and derivatives such as o-coumaric acid, *trans*-2-phenylcyclopropane-1-carboxylic-acid, *trans*-2-methylcinnamic acid, and *trans*-2-methoxycinnamic acid as well as synthetic compound ethyl 2-nitro-3-arylacrylates, have been reported to affect the expression of *X. oryzae* T3SS genes (Fan et al., 2017; Jiang et al., 2019). Khokhani et al. (2013) found T3SS inhibitors (4-methoxy-cinnamic acid and benzoic acid) and T3SS inducers [*trans*-2-(4-hydroxyphenyl)-ethenylsulfonate] change the expression of *E. amylovora* T3SS through HrpS-HrpL pathway, and salicylic acid (SA) inhibits *hrpA* promoter activity. More recently, salicylidene acylhydrazide derivatives, which were described as inhibitors of T3SS in zoonoses, have been verified for their effects on management of plant diseases caused by *Ralstonia solanacearum* and *P. syringae* pv. *tomato* (Puigvert et al., 2019). In addition, plant-derived compounds eugenol, SA, chlorogenic acid, resveratrol, CA, and fumaric acid were reported as inducers of T3SS in *R. solanacearum* (Joshi et al., 2021), p-hydroxybenozic acid (PHBA) and vanillic acid have been identified as inhibitors of T3SS transcription in *P. syringae* pv. *tomato* (Kang et al., 2020).

In this study, we selected some plant natural compounds for screening of *Dickeya* T3SS inhibitors using a *hrpA-gfp* bioreporter by Multifunctional Microplate Reader, followed by flow cytometer. The compounds significantly repressed the *hrpA* promoter activity without affecting bacterial growth, were used for in-depth evaluation on their potential on prevention of bacterial soft rot of important crops.

## Materials and Methods

### Bacterial Strains, Plasmids, and Growth Conditions

The bacterial strains, plasmids, and primers used in this study are listed in Supplementary Table S1. *Dickyea* bacterial strains including *D. zeae* MS2 isolated from banana (Hu et al., 2018; Feng et al., 2019), *D. oryzae* EC1 isolated from rice (Zhou et al., 2011; Cheng et al., 2013; Lv et al., 2019), *D. dadantii* 3937 isolated from *Saintpaulia ionantha* (Kotoujansky et al., 1982; Boucher et al., 1987; Glasner et al., 2011), and *D. fangzhongdai* CL3 isolated from taro, were grown in LB or LS5 medium at 28°C. LS5 is a nutrient poor medium (Liao et al., 2014), which induces *hrp* gene expression. *Escherichia coli* and the derived strains were grown in LB medium at 37°C.

To construct the reporter strain of *hrpA* promoter, a fragment containing the promoter region of *hrpA*, harboring a putative *hrp*-box (GGAACCATCTCTTGCTATCTCCTACTTA), was amplified using the primer pair PhrpA-F-*Hind*III (5′-ggaattggggatcggaagcttCTGGCCCGGCAACATCCGT-3′) and PhrpA-R-*Bam*HI (5′-gagctcggtacccggggatccGCAACTTCATGCTATCCATAG-3′; Supplementary Figure S1) with MS2 genomic DNA as the template. The fragment was purified using NucleoSpin® Gel and PCR Clean-up kit (MACHEREY-NAGEL GmbH & Co. KG, Düren Neumann Neander, Germany) and ligated with the *Hind*III/*Bam*HI-digested pPROBE-NT plasmid (Miller et al., 2000) using ClonExpress® MultiS kit (Vazyme Biotech Co., Nanjing, China). The resultant plasmid was verified by DNA sequencing and transformed into the strain MS2 by triparental conjugation (Zhou et al., 2016), which was designated as MS2 (pPhrpA-gfp).

To generate *gacA* deletion mutants, upstream and downstream fragments of the deleted genes were respectively amplified using primers gacA-1 & gacA-2, and gacA-3 & gacA-4 listed in Supplementary Table S1, and purified with NucleoSpin Gel and PCR Clean-up kit. The fragments were fused with the *Bam*HI and *Spe*I digested suicide plasmid pKNG101 using ClonExpress® MultiS kit (Vazyme Biotech Co., Nanjing, China). The construct was transformed into *E. coli* CC118λ competent cells and introduced into strain MS2 by triparental conjugation using the method described previously (Zhou et al., 2016). The resultant *gacA* deletion mutant was confirmed by PCR using the primer pair gacA-F & gacA-R.

Antibiotics were added at the following final concentrations when required: kanamycin (Km), 50 μg/ml; and streptomycin (Sm), 50 μg/ml.

### Sources of the Screened Compounds

The compounds used in this work are listed in Table 1. All compounds were purchased from Guangzhou Dingguo Biotechnology Co. LTD, and dissolved in dimethyl sulfoxide (DMSO) to corresponding concentrations.

**Table 1:** Screening for inhibitors of *Dickeya zeae* MS2 type III secretion system (T3SS) by Multifunctional Microplate Reader.

ID	Compound (0.2 mM)	Chemical structures	Average MFI ± SD^a^	DMSO%^b^
**Phytohormone**				
IAA	Indole-3-acetic acid	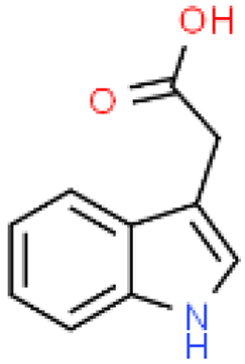	11,608.67 ± 137.42	96.58
MeJA	Methyl Jasmonate	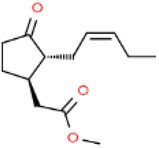	12,568.33 ± 248.82	104.56
SA	Salicylic acid	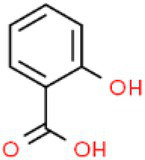	4,962.33 ± 77.69	41.28
GA3	Gibberellin A3	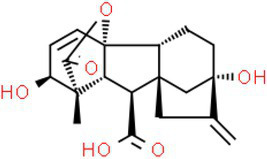	11,248.33 ± 178.20	93.58
t-Z	Trans-zeatin	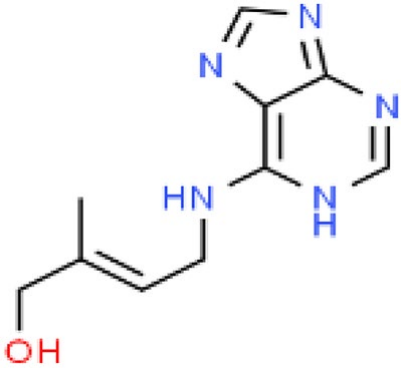	11,491.33 ± 436.43	95.60
**Other compounds**				
HA	Hydrocinnamic acid	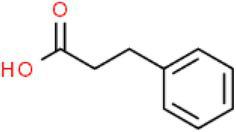	8,073.00 ± 87.43	67.16
PHBA	p-Hydroxybenzoic acid	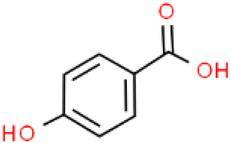	8,868.00 ± 62.07	73.77
OCA	o-Coumaric acid	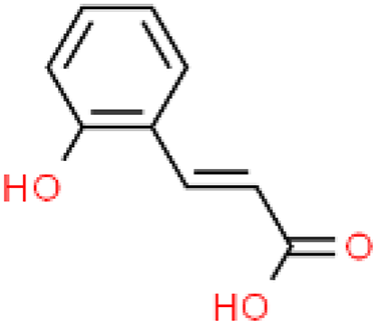	11,441.33 ± 170.00	95.18
PCA	p-Coumaric acid	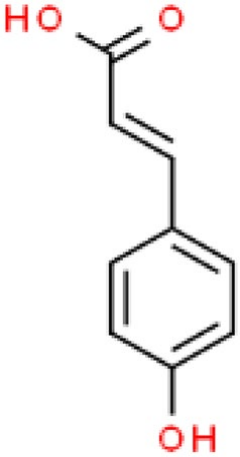	4,783.00 ± 168.59	39.79
2-CCA	2-Chlorocinnamic acid	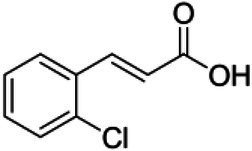	11,108.33 ± 85.23	92.41
CA	Cinnamic alcohol	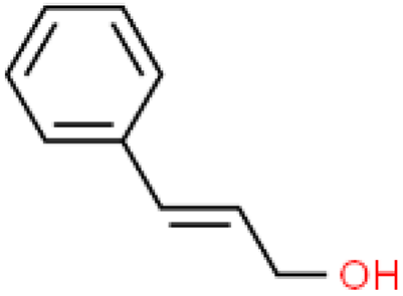	6,198.00 ± 355.33	51.56
AS	Acetosyringone	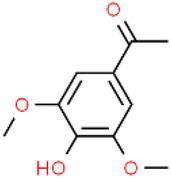	17,213.00 ± 217.44	143.20

Average MFI ± SD of dimethyl sulfoxide (DMSO) was 12,020.33 ± 226.40.

a *Dickeya zeae* MS2 cells carrying the green fluorescent protein (GFP) reporter pPhrpA were grown in LS5 supplemented with 0.2 mM compounds for 14 h. GFP mean fluorescence intensity (MFI) of bacterial cells was determined by Multifunctional Microplate Reader. Three replicates were used in each compound and three independent experiments were performed.

b DMSO% represents the relative promoter activity of hrpA, calculated according to the formula: DMSO% = 100 × MFI_compound_/MFI_DMSO_.

### Detection of the Mean Fluorescence Intensity of *hrpA* Promoter Activity

MS2(pPhrpA-gfp) were cultured in LB medium supplemented with Km to OD_600_ of 2.0 by shaking at 200 rpm at 28°C, then transferred into LS5 medium added with kanamycin and 0.2 mM each tested compound listed in Table 1 at a ratio of 1:10, 100 μl of which was added into each well of polystyrene 96 well-tissue culture plate (Guangzhou Jet Bio-Filtration Co., Ltd., China). Three replicates were set for each culture. The plate was then placed in an incubator at 28°C and shaking-cultured in the dark at 200 rpm for 14 h. The fluorescence intensity and optical density (OD) of each well was detected by a Multifunctional Microplate Reader (Microplate reader, BioTek, United States). The excitation and emission wavelength of fluorescence intensity at 485 and 528 nm, respectively. The experiment was repeated three independent times. Equal volume of DMSO and LS5 medium was respectively used as the solvent control and blank control. The fluorescence intensity of LS5 medium supplemented with the compounds at the concentration of 0.2 mM was compared with that of LS5 medium and LS5 + DMSO to test whether individual compounds might have their own fluorescence.

To verify the screened inhibitor candidates, the *hrpA* promoter activity of the reporter strain was evaluated under corresponding concentrations by flow cytometer. Briefly, MS2(pPhrpA-gfp) was cultured in LB medium to OD_600_ of 2.0 at 28°C, and then transferred into LS5 medium containing the corresponding concentration of each compounds at a ratio of 1:100 and shaking-cultured in the dark at 200 rpm, 28°C for 14 h. Equal volume of DMSO was used as the solvent control. Three replicates were conducted for each compound treatment. Bacterial cells were resuspended in 1 × phosphate-buffered saline (PBS) to OD_600_ of 0.3. The fluorescence intensity of each treatment was detected by FACS-Caliber flow cytometer (CytoFLEX, Brea, CA, United States).

### Measurement of *Dickeya zeae* MS2 Growth Treated With the Five Screened Compounds

To determine whether the screened compounds with effects on *hrpA* promoter activity have impacts on the growth of *D. zeae* MS2, we measured the optical densities of MS2 treated with different concentrations of the five compounds in nutrient-limited LS5 medium (used to measure the induced T3SS expression) using Bioscreen automatic growth curve analyzer (Bioscreen, Finland). Briefly, MS2 single colony was cultured in LB medium to OD_600_ of 2.0 at 28°C by shaking at 200 rpm, then transferred into LS5 medium added with the different concentrations of the tested compounds or DMSO at a ratio of 1:10, and 200 μl of which was dispended into each well of a 96 well-tissue culture plate. Growth of the bacterium was measured every 2 h at 600 nm for 44 h. At the same time, colony forming units (CFU) were counted by gradient dilution of the bacterial suspension at 14 h to analyze the effects of five compounds on bacterial growth. On each LB agar plate, 100 μl bacterial diluent was evenly spread and cultured at 28°C for 24 h. Plates with colony numbers between 30 and 300 were selected to count the CFU. The wide-type MS2 was used as the positive control. Three replicates were used in each compound and three independent experiments were performed.

### Non-host Tobacco Plant Hypersensitive Response Assay

MS2 cells were grown in LB medium and shaking cultured at 200 rpm at 28°C to OD600 of 2.0, and then transferred into 10 ml of LS5 medium at a ratio of 1:100, shaking cultured till OD_600_ of 0.6. The bacterial cells were collected and suspended in LS5 medium added with optimal concentrations of the tested compounds or DMSO for incubation in the dark at 28°C for 4 h before leaf infiltration. *Nicotiana tobacum* K326 plants were used for HR assays (Hu et al., 2022), where 100 μl (4.8 × 10^6^ CFU) of bacterial suspensions were press-infiltrated to tobacco leaves using 1.0 ml needleless syringes. HRs were photographed at 24 h. The area of lesions was measured using ImageJ software. Three replicates were used in each compound and three independent experiments were performed. Equal volume of MS2 + DMSO and LS5 + DMSO was used as the positive and negative control, respectively.

### RNA Extraction and qRT-PCR Analysis

MS2 cells were cultured in LB medium to OD_600_ of 1.5 at 28°C by shaking at 200 rpm, and then transferred into LS5 medium containing optimal concentration of each compound at a ratio of 1:100 for shaking culture in the dark at 200 rpm at 28°C to OD_600_ of 0.8. Total MS2 RNA was isolated using SV total RNA isolated system kit (Promega, Madison, WI, United States), further purified using RNA clean kit (Qiagen, Hilden, Germany), and treated with DNaseI to degrade any possible DNA contamination, and then 1 μg of RNA was reverse transcribed using HiScriptII Q RT SuperMix Kit (Vazyme Biotech Co., Nanjing, China). The cDNA levels of different samples were quantified by real-time PCR (RT-PCR) using a SYBR Green Master Mix (Vazyme Biotech Co., Nanjing, China). For quantitative PCR, the cDNA template was diluted 40 times and 1 μl of cDNA was added to 20 μl of reaction system. Primer efficiency (between 90% and 107%) of each gene was determined using DNA standards at different concentrations. To calculate the relative expression level of target genes, the expression level of a housekeeping gene *atpD* was used as the internal control. The relative levels of gene expression were determined using the 2^-ΔΔCT^ method (Livak and Schmittgen, 2001). All gene expression under compound treatment or no solvent (MS2) was compared with that of MS2 + DMSO using Student’s *t*-test analysis. Three technical replicates were used each time. The procedure for analysis on the expression of *rpoN*, *hrpL*, and *gacA* in wild-type MS2 and Δ*gacA* mutant was the same as above except that the strains were grown in LB medium. The expression of *gacA* in ΔgacA was used as a negative control. The primers of quantitative real-time PCR (qRT-PCR) are listed in Supplementary Table S1.

### Pathogenicity Tests on Host Plants

To evaluate the efficacy of the five screened inhibitors on restraining the virulence or infection of *Dickeya*, different *Dickeya* bacteria including *D. zeae* MS2, *D. oryzae* EC1, *D. dadantii* 3937, and *D. fangzhongdai* CL3, were grown overnight in LB medium with shaking at 200 rpm at 28°C and then diluted into 10 ml of fresh LS5 at a ratio of 1:100 and grown to OD_600_ of 1.0. The bacterial cells were collected and suspended in LS5 medium supplemented with optimal concentrations of the tested compounds or DMSO. The cell suspensions were incubated in the dark at 28°C for 4 h before plant inoculation. Equal volume of MS2 + DMSO and LS5 + DMSO was used as the positive and negative control, respectively.

For inoculation on dicotyledonous potato and monocotyledonous taro, tubers were sliced in 5 mm-thickness and dried for about 20 min at room temperature. An aliquot of 2 μl (9.6 × 10^4^ CFU) of *D. dadantii* 3937 bacterial cells were then inoculated on the center of potato slices, and 5 μl (2.4 × 10^5^ CFU) of CL3 bacterial cells were inoculated on the center of taro slices. After inoculation, tissue slices were placed in a growth chamber with conditions of 28 ± 2°C and 75 ± 15% relative humidity for about 24 h. Image J software was used to measure the area of lesions.

To test the effect of the five compounds on controlling rice foot rot disease, eight rice seedlings were planted in each pot and grown for 3 weeks. Firstly, a 1.0 ml sterile needle syringe was used to stab the roots of the seedlings, 175 ml (8.4 × 10^9^ CFU) of the EC1 and inhibitor mixture (incubated for 4 h) was poured into the pot. All pots were placed in the growth chamber with conditions of 28°C and 95% relative humidity with 12 h alternating light–dark cycles for 3 days. Three replicates were used in each compound and three independent experiments were performed. Equal volume of LS5 liquid medium was used as the blank control.

For inoculation on banana (*Musa* ABB), 3~4 leaf seedlings were selected and acclimated for 2 weeks prior at 25°C with 12 h alternating light–dark cycles before inoculation. MS2 bacterial suspensions in 500 μl (2.4 × 10^7^ CFU) were injected into the center of banana pseudostem using a 1.0 ml needleless syringe. Plants were placed in the growth chamber with conditions of 28°C and 95% relative humidity with 12 h alternating light–dark cycles for 7 days. The severity of disease in rice and banana was assessed using the virulence scoring method described in our previous study (Feng et al., 2019; Hu et al., 2022).

### Statistical Analysis

GraphPad Prism 8.4.3 software was used for statistical analysis. The results were analyzed by Student’s *t*-test.

## Results

### Identification of Small Molecule Compounds That Inhibit or Induce the *hrpA* Promoter Activity of MS2

As we all know, new drugs targeting T3SS that can block its function without affecting the growth and survival of bacteria have been found in many bacteria, which undoubtedly becomes a new strategy for the prevention and treatment of bacterial diseases. To screen T3SS inhibitors or inducers of *D. zeae*, a promoter-*gfp* fusion plasmid was firstly constructed, which includes a promoter of *hrpA* of *D. zeae* banana strain MS2 (Hu et al., 2018; Feng et al., 2019), a green fluorescent protein (GFP) encoding gene and a kanamycin resistance gene. Secondly, the resultant plasmid pPhrpA-gfp was transformed into the MS2 parental cells and grown in T3SS-inducing LS5 medium supplemented with each tested compound at a concentration of 0.2 mM for 14 h. Next, the mean fluorescence intensity (MFI) representing the promoter activity of *hrpA* was detected by microplate reader, and the compounds that impact the promoter activity of *hrpA* were preliminarily screened out. Among the compounds screened, five compounds at a concentration of 0.2 mM showed obvious inhibitory effect on *hrpA* promoter activity, which are SA, p-hydroxybenzoic acid (PHBA), CA, PCA, and hydrocinnamic acid (HA), respectively (Table 1). These five compounds are not self-fluorescent at the concentration of 0.2 mM (Supplementary Figure S2).

**Figure 1:**
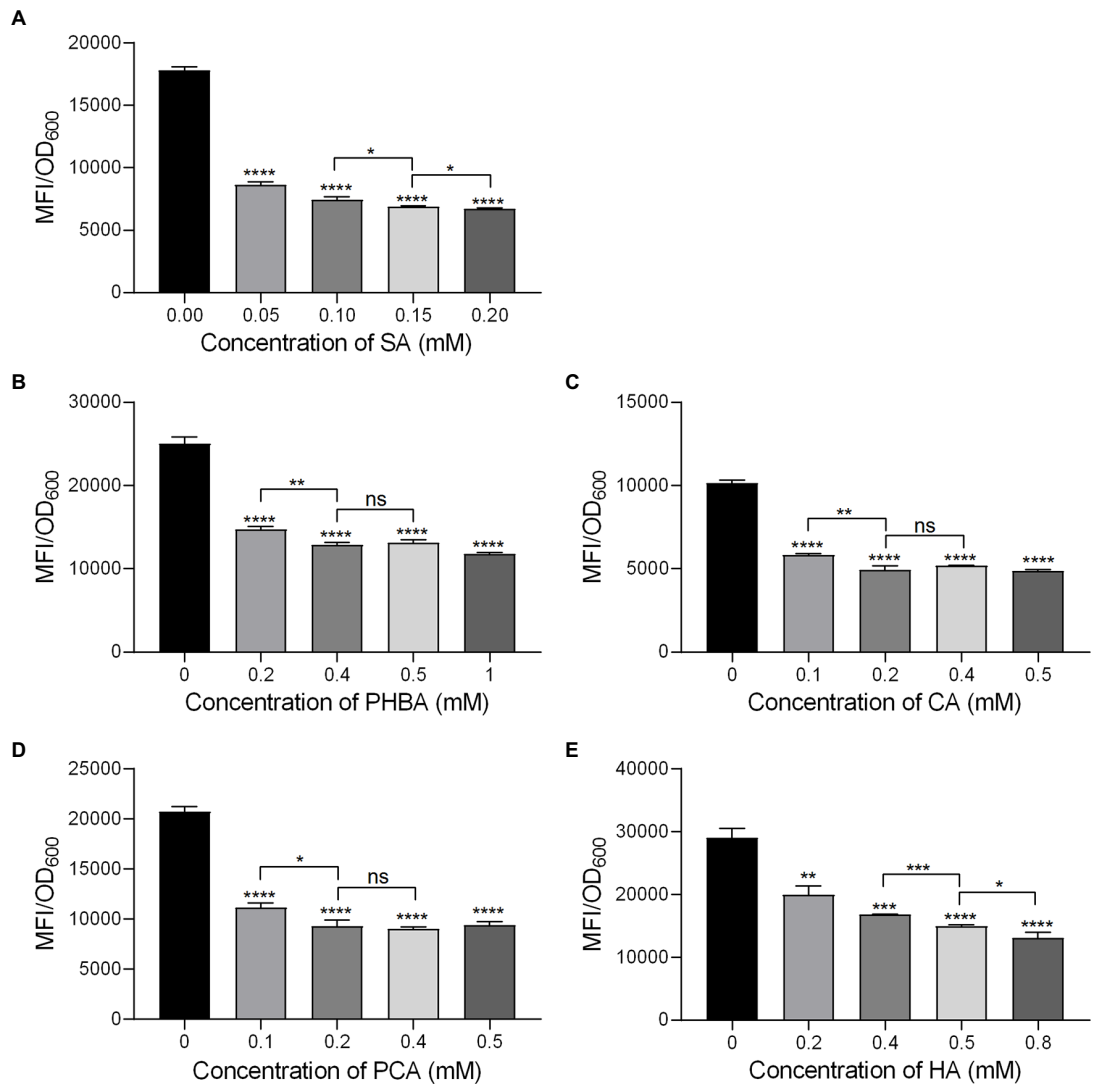
The *hrpA* promoter activity of MS2 in LS5 medium supplemented with different concentrations of salicylic acid (SA; **A**), p-hydroxybenzoic acid (PHBA; **B**), cinnamyl alcohol (CA; **C**), p-coumaric acid (PCA; **D**), and hydrocinnamic acid (HA; **E**) at 14 h of growth. MS2(pPhrpA-gfp) cultures (OD_600_ of 2.0) were transferred into the LS5 medium supplemented with different concentrations of tested compounds or DMSO at a ratio of 1:10, and then dispended into a 96 well-tissue culture plate (100 μl per well) for cultivation in the dark for 14 h. MFI was measured by a Multifunctional Microplate Reader. DMSO was used as the positive control. Three replicates were used in each compound and three independent experiments were performed with similar results. For statistical analysis, GraphPad Prism 8.4.3 software was used to perform Student’s *t*-test. Asterisks indicate statistically significant differences (^*^p < 0.05, ^**^p < 0.01, ^***^p < 0.001, and ^****^p < 0.0001).

In order to determine the optimal concentration of the compounds to inhibit the *hrpA* promoter activity, the MFI of MS2(pPhrpA-gfp) treated with different concentrations of the five compounds was detected by microplate reader. The results showed that with the increase of concentration, the MFI value of MS2(pPhrpA-gfp) under the treatment of the five compounds decreased to different degrees (Figure 1). For compounds SA and HA, concentrations of 0.2 and 0.8 mM, respectively achieved strongest inhibitory activity of *hrpA* promoter in their corresponding assay (Figures 1A,E), but could affect the growth of MS2(pPhrpA-gfp) under these two concentrations (Supplementary Figure S3). About 0.2 mM PCA slightly promoted bacterial growth (Supplementary Figure S3). Therefore, the optimal concentrations of the five compounds acting on the *hrpA* promoter activity at 14 h of bacterial growth were determined as 0.15 mM SA, 0.4 mM PHBA, 0.2 mM CA, 0.2 mM PCA, and 0.5 mM HA (Figure 1; Supplementary Figure S3), which were used for the following study. Then the five compounds at respective optimal concentration were measured for their alterations in *hrpA* promoter activity through the Multifunctional Microplate Reader and FACS-Caliber flow cytometer, the results showed that compared with the DMSO control, the MFI decreased to less than 46.93 and 36.96%, respectively, and the inhibitory rate of *hrpA* promoter activity was more than 53.07 and 63.04%, respectively (Table 2), indicating that all the five compounds could significantly inhibit the *hrpA* promoter activity.

**Table 2:** The *hrpA* promoter activity of MS2 in LS5 medium supplemented with optimal concentrations of compounds at 14 h of growth and the corresponding inhibition rates of the compounds toward *hrpA* promoter activity.

	Multifunctional Microplate Reader			Flow cytometer		
Compound	MFI ± SD^a^	DMSO%^b^	Inhibition rate% (100%-DMSO%)	MFI ± SD^a^	DMSO%^b^	Inhibition rate% (100%-DMSO%)
DMSO	16,605.33 ± 75.61	/	/	11,520.60 ± 349.49	/	/
0.15 mM SA	6,660.67 ± 35.16	40.11	59.89	2,201.97 ± 177.32	19.11	80.89
0.4 mM PHBA	7,792.67 ± 108.04	46.93	53.07	4,258.57 ± 251.78	36.96	63.04
0.2 mM CA	7,082.00 ± 55.46	42.65	57.35	4,005.77 ± 100.93	34.77	65.23
0.2 mM PCA	6,569.00 ± 84.55	39.56	60.44	2,622.47 ± 154.13	22.76	77.24
0.5 mM HA	7,549.67 ± 59.53	45.47	54.53	2,988.77 ± 118.56	25.94	74.06

a *Dickeya zeae* MS2 cells carrying the GFP reporter pPhrpA were grown in LS5 supplemented with optimal concentration of each compound for 14 h. GFP MFI of bacterial cells was determined by Microplate Reader and Flow Cytometer. Three replicates were used in each compound and three independent experiments were performed.

b DMSO% represents the relative promoter activity of hrpA, calculated according to the formula: %DMSO = 100 × MFI_compound_/MFI_DMSO_.

### Growth of MS2 Is Not Affected by the Five Tested Compounds at Respective Optimal Concentrations

**Figure 2:**
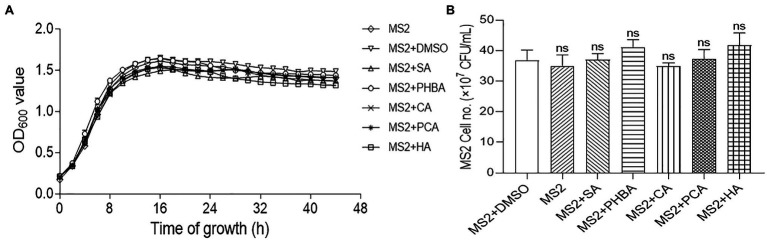
Effects of SA (0.15 mM), PHBA (0.4 mM), CA (0.2 mM), PCA (0.2 mM), and HA (0.5 mM) on the growth of MS2 in LS5. **(A)** The growth curves of MS2 in LS5 supplemented with DMSO or tested compounds, respectively. MS2 culture (OD_600_ of 2.0) was transferred into LS5 medium supplemented with candidate concentrations of tested compounds at a ratio of 1:10, and then dispended into a 96 well-tissue culture plate (200 μl per well). Optical density (OD_600_) of bacterial cultures was recorded every 2 h during a 44-h period using a Bioscreen automatic growth curve analyzer. Each point indicates the average value of three replicates with error bars representing the SD. **(B)** Colony forming units (CFU) of MS2 at 14 h of growth in LS5 supplemented with DMSO or tested compounds, respectively. Bacterial suspensions were diluted in gradient, 100 μl of which was spread on LB agar plate and cultured at 28°C for 24 h. Plates with colony numbers between 30 and 300 were selected to count the CFU. MS2 + DMSO was used as the positive control. The data were the mean of three replicates and subjected to Student’s *t*-test analysis by Graphpad Prism 8.4.3 (ns, no statistical significance).

To exclude the possibility that the decrease in MFI was due to the influence of the compounds on bacterial growth, we tested the effects of the compounds on the growth of *D. zeae* MS2. Firstly, the growth rate of MS2 in LS5 medium supplemented with each of the compounds at optimal concentration or DMSO was measured by Bioscreen automatic growth curve analyzer. Results showed that compared with the untreated control, addition of either DMSO or compounds SA, PHBA, CA, PCA, or HA at their respective optimal concentration had no obvious impact on MS2 growth rate at different time points (Figure 2A). Secondly, the live bacterial cells of MS2 were also counted at 14 h of bacterial growth. Similar result was obtained that the five compounds had no significant effect on the CFU of MS2 compared with the medium or DMSO control (Figure 2B), indicating that the five compounds at respective optimal concentration do not affect the normal growth and survival of *D. zeae* MS2.

### The Five Compounds Suppress HR Caused by MS2 on Tobacco

**Figure 3:**
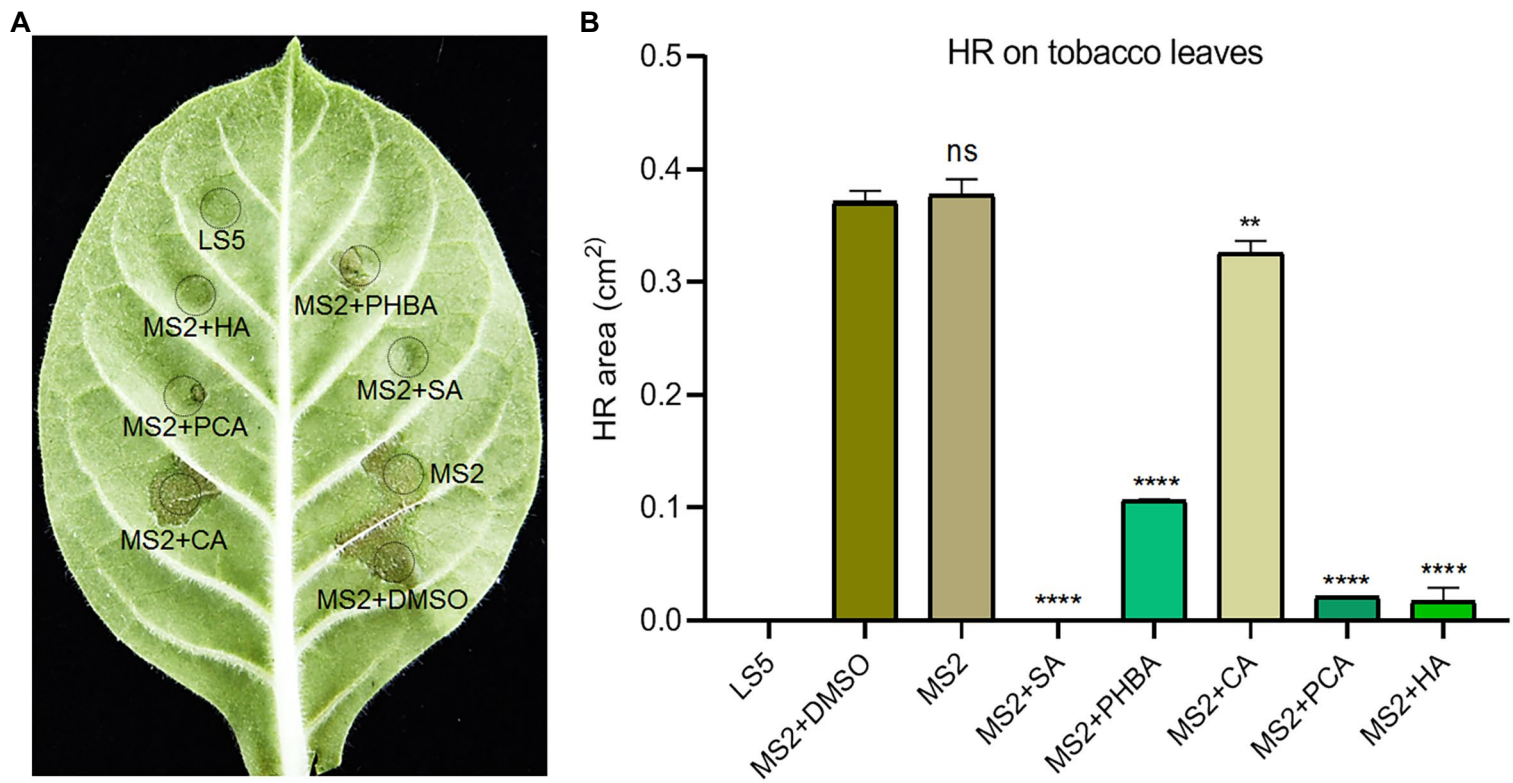
Effects of SA (0.15 mM), PHBA (0.4 mM), CA (0.2 mM), PCA (0.2 mM), and HA (0.5 mM) on the hypersensitive response (HR) induced by MS2 on tobacco leaves. **(A)** HR symptoms on *Nicotiana tabacum* K326 leaves. Cell suspensions of MS2 at OD_600_ of 0.6 were incubated in the dark with optimal concentrations of the tested compounds or DMSO for 4 h, then 100 μl of bacterial suspensions were press-infiltrated to tobacco leaves. HRs were photographed at 24 h. **(B)** The area of HR lesions. ImageJ software was used to measure the area of lesions. MS2 + DMSO was used as the positive control. The data were the mean of three replicates and subjected to Student’s *t*-test analysis by Graphpad Prism 8.4.3 (ns, no statistical significance, ^**^p < 0.01, ^****^p < 0.0001).

MS2 cells with functional T3SS can induce HR on non-host tobacco leaves. To test the influence of the five compounds on the elicitation of HR, we added the above five compounds to the MS2 suspensions at their respective optimal concentration for incubation for 2 h and then infiltrated them into tobacco leaves using sterile 1.0 ml needleless syringes. The MS2 or MS2 suspended in DMSO triggered visible HR symptoms on tobacco leaves after 24 h post inoculation (hpi), when addition of the five compounds significantly suppressed the HR reaction on tobacco leaves (Figure 3). Specifically, 0.15 mM SA completely inhibited the HR of MS2 on tobacco, followed by 0.5 mM HA and 0.2 mM PCA with up to 94 and 95% inhibition effect on HR, respectively (Figure 3). Among the five compounds, 0.2 mM CA got the weakest inhibition effect on HR response induced by MS2 (Figure 3).

### Inhibitory Effect of the Five Compounds on the Transcription of T3SS-Related Genes

**Figure 4:**
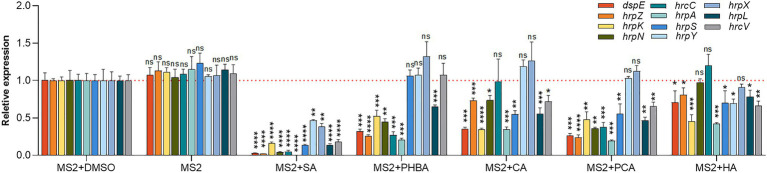
Effects of SA (0.15 mM), PHBA (0.4 mM), CA (0.2 mM), PCA (0.2 mM), and HA (0.5 mM) on the expression of T3SS-related genes in MS2. MS2 cells were cultured in LS5 medium added with optimal concentrations of the tested compounds or DMSO. RNA was collected at OD_600_ of 0.8. The cDNA levels of different samples were quantified by real-time PCR (RT-PCR) using a SYBR Green Master Mix. A housekeeping gene *atpD* was used as an endogenous control for data analysis. All gene expression under compound treatment or no solvent (MS2) was compared with that of MS2 + DMSO using Student’s *t*-test analysis (Graphpad Prism 8.4.3). Three independent tests were performed with similar results (ns, no statistical significance, ^*^p < 0.05, ^**^p < 0.01, ^***^p < 0.001, and ^****^p < 0.0001).

**Figure 5:**
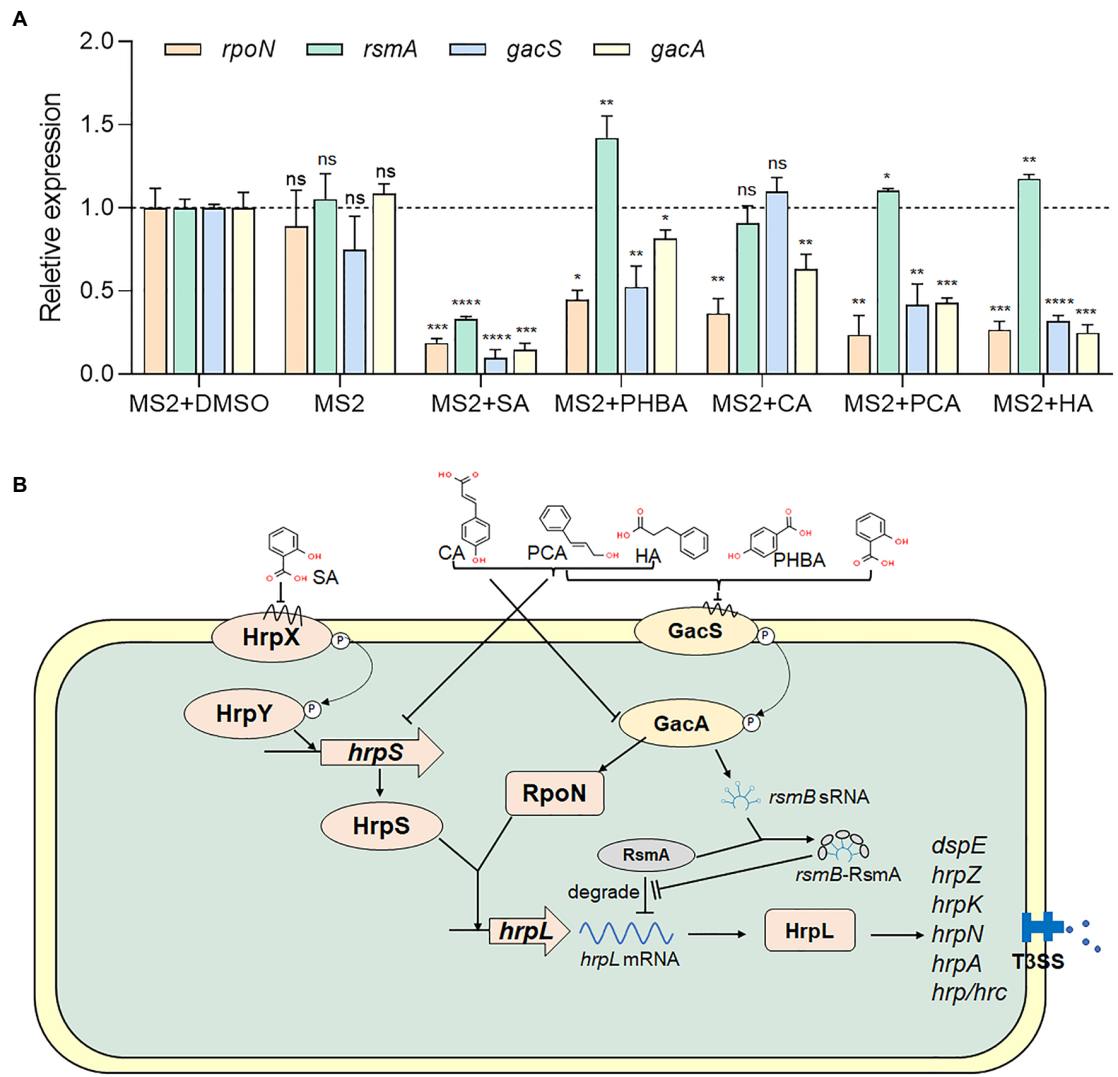
Regulation of SA, PHBA, CA, PCA, and HA on T3SS. **(A)** Effects of SA (0.15 mM), PHBA (0.4 mM), CA (0.2 mM), PCA (0.2 mM), and HA (0.5 mM) on the expression of *rpoN*, *rsmA*, *gacS*, and *gacA*. RNA was collected at a bacterial concentration of OD_600_ of 0.8. The cDNA levels of different samples were quantified by RT-PCR using a SYBR Green Master Mix. A housekeeping gene *atpD* was used as an endogenous control for data analysis. All gene expression under compound treatment or no solvent (MS2) was compared with that of MS2 + DMSO using Student’s *t*-test analysis (Graphpad Prism 8.4.3). Three independent tests were performed with similar results (ns, no statistical significance, ^*^p < 0.05, ^**^p < 0.01, ^***^p < 0.001, and ^****^p < 0.0001). **(B)** Regulatory network controlling the *Dickeya zeae* T3SS. In *Dickeya* bacteria, the expression of T3SS is regulated by a master regulator, HrpL (Hu et al., 2022). On one hand, *hrpL* upregulates many *hrp* genes that encode the T3SS structural and functional proteins, such as *hrpA*, *hrpN*, and *dspE* (Hu et al., 2022). On the other hand, the expression of *hrpL* is regulated by the HrpX/HrpY-HrpS-HrpL pathway at the transcriptional level and the GacS-GacA-RsmB-RsmA pathway at the post-transcriptional level (Chatterjee et al., 2002; Yap et al., 2005; Yuan et al., 2020). ⊥, negative control; →, positive control.

To understand whether the five screened compounds have inhibitory effects on the expression of T3SS-related genes of MS2, qRT-PCR was performed to measure relative transcriptional levels of some representative genes in the *dsp*/*hrp*/*hrc* gene clusters. The qRT-PCR results demonstrated that, compared with the MS2 + DMSO control, the expression levels of many T3SS tested genes were significantly altered when MS2 was incubated with the optimal dosage of each of the five molecules (Figure 4). In the presence of 0.15 mM SA, the mRNA expression levels of all the tested T3SS-related genes were significantly decreased, and in the presence of 0.4 mM PHBA, 0.2 mM CA, 0.2 mM PCA, or 0.5 mM HA, the transcription level of partial T3SS genes was significantly lower than that of the solvent control (Figure 4). It is worth noting that the mRNA level of *hrpL* was reduced by more than 35% in the presence of four inhibitors (SA, PHBA, CA, and PCA), in which, 0.15 mM SA reduced the mRNA level of *hrpL* by approximately 90%. HA in 0.5 mM slightly affected the expression of *hrpL*, and dramatically reduced the mRNA expression level of *hrpA* (Figure 4), suggesting that HA may directly actions on the *hrpA*. Our results indicated that SA, PHBA, CA, and PCA suppress T3SS gene expression probably through the T3SS master regulator HrpL. Current investigation about the regulation of T3SS reveals that the expression of HrpL is regulated by several regulatory pathways including the GacS/GacA-RsmB-RsmA pathway and the σ^54^-containing RNA polymerase holoenzyme RpoN, apart from the HrpX/HrpY-HrpS-RpoN pathway (Chatterjee et al., 2002; Yap et al., 2005; Yuan et al., 2020). qRT-PCR analysis indicated that the expression of *rpoN* and *gacA* is regulated by all of these five compounds in consistent pattern, while the expression of *rsmA* is only downregulated by 0.15 mM SA (Figure 5A). To determine whether there is any interaction between RpoN and GacA, qRT-PCR analysis was performed to measure the *rpoN* and *gacA* expression in the ΔgacA mutant (ΔrpoN was not obtained in this study). Results showed that mutation of *gacA* resulted in decreased expressions of the *rpoN* by 1.96-fold, which indicates that GacA positively regulates *rpoN* (Supplementary Figure S4). Similarly, we examined the *hrpL* expression and found that the transcript level of *hrpL* was downregulated by 4.2-fold in the ΔgacA mutant (Supplementary Figure S4). Thus, the mechanism mode for the T3SS-inhibiting compounds was drawn as Figure 5B.

### The Five T3SS Inhibitors Have Good Performance on Alleviating Crop Soft Rot Caused by *Dickeya*

**Figure 6:**
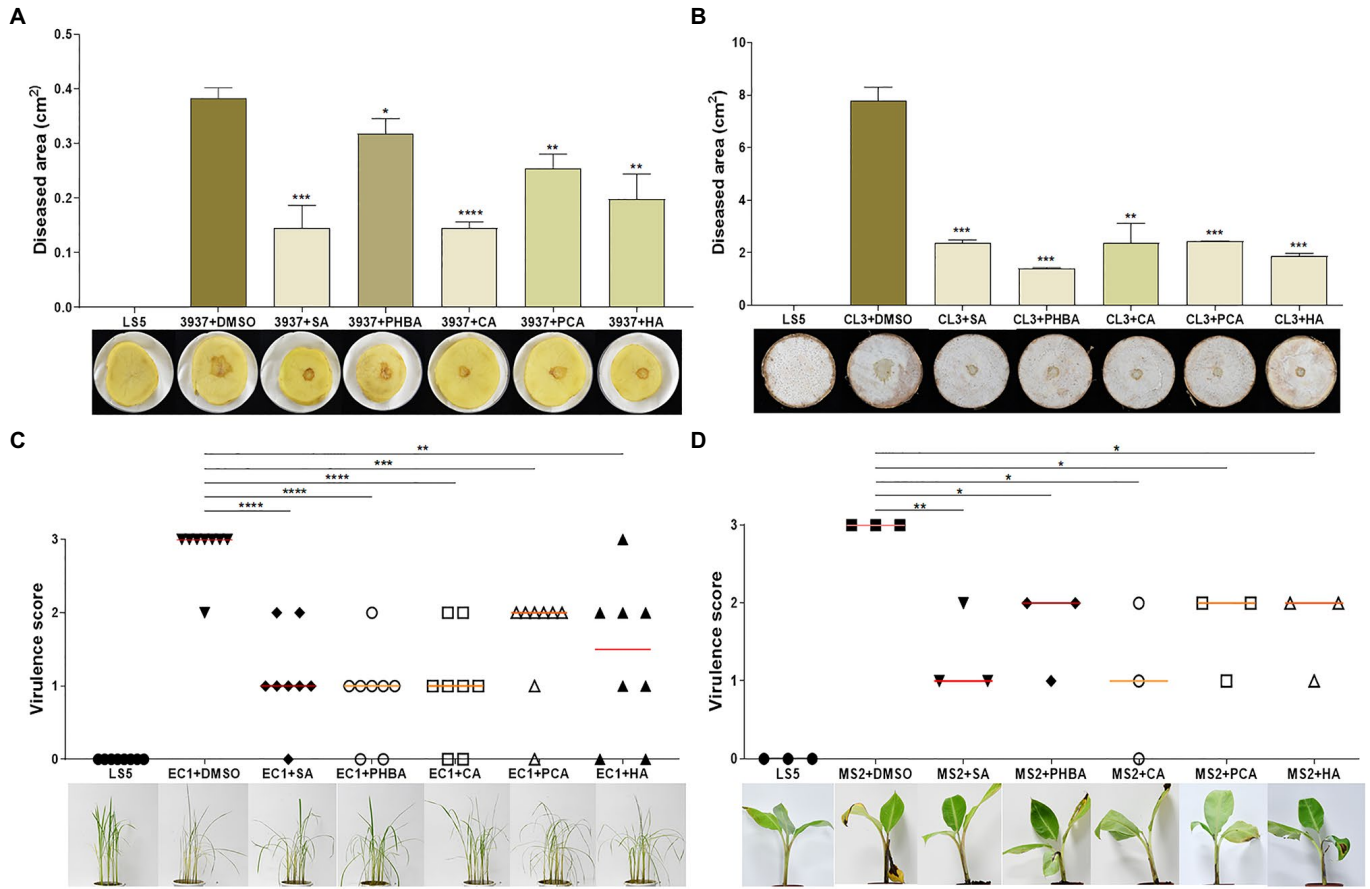
Inhibition of SA (0.15 mM), PHBA (0.4 mM), CA (0.2 mM), PCA (0.2 mM), and HA (0.5 mM) against crop soft rot disease on potato **(A)**, taro **(B)**, rice **(C)**, and banana **(D)**. Cell suspensions of *Dickeya dadantii* 3937, *D. fangzhongdai* CL3, *D. oryzae* EC1, and *D. zeae* MS2 at OD_600_ of 1.0 were incubated in the dark with optimal concentrations of the tested compounds or DMSO for 4 h. About 2 μl (9.6 × 10^4^ CFU) of *D. dadantii* 3937 and 5 μl (2.4 × 10^5^ CFU) of CL3 bacterial cells were respectively applied on the center of potato and taro slices, 500 μl (2.4 × 10^7^ CFU) of MS2 bacterial cells were injected into the pseudostems of banana seedlings, and 175 ml (4.8 × 10^9^ CFU) of EC1 bacterial cells were irrigated into pots with rice seedlings after needle punctures on stem bases. Three potato slices, taro slices, and banana seedlings, and eight rice seedlings were inoculated each time, and three independent tests were performed with similar results. MS2 + DMSO was used as the positive control. The data were subjected to Student’s *t*-test analysis by Graphpad Prism 8.4.3 (ns, no statistical significance, ^*^p < 0.05, ^**^p < 0.01, ^***^p < 0.001, and ^****^p < 0.0001).

To test whether the above five T3SS inhibitors could affect the ability of the *Dickeya* to induce disease symptoms in soft rot diseases, we incubated the five compounds with several *Dickeya* pathogens isolated from different sources for 4 h and then inoculated them into dicotyledonous potato slices, and monocotyledonous banana and rice seedlings as well as taro slices. After 24 hpi, the potato and taro slices inoculated with *Dickeya* bacteria supplemented with the five T3SS inhibitors at their optimal concentration significantly reduced soft rot disease symptoms compared with those inoculated with controls of *Dickeya* + DMSO (Figures 6A,B).

We further investigated the effect of *D. oryzae* EC1 on rice seedlings after treatment with five compounds. On EC1 + DMSO-infected rice, apparent disease symptoms were observed after 3 days, including the base of the stem appeared brown, the middle stem and leaves turned yellow and the leaves showed signs of water loss and wilting (Figure 6C). However, these symptoms were significantly reduced by treatment of the bacterial germs with five inhibitors (Figure 6C). Additionally, bacterial suspensions treated with compounds were injected into the center of banana pseudostem using a 1.0 ml syringe. After a week of incubation, the results showed that compared with the control groups, the soft rot symptoms caused by MS2 on banana seedlings were alleviated and weakened (Figure 6D).

## Discussion

*Dickeya* is one of the top 10 important bacterial phytopathogens in the world, and the bacterial soft rot caused by *Dickeya* spp. often results in serious economic losses to crop yields, especially on rice, potato, banana, and taro (Mansfield et al., 2012; van der Wolf et al., 2014; Zhang et al., 2014; Li et al., 2020; Huang et al., 2021). Plant molecules play an important role in host–microbe interaction. An increasing number of studies have proven that plant natural compounds such as cinnamaldehyde, cinnamic, coumaric acid, SA, carvacrol, syringic, and catechol *via* many virulence pathways like quorum sensing (QS), T3SS, motility, biofilm formation, and exoenzyme activity directly affect pathogenicity of *Pectobacterium* and/or *Dickeya* (Li et al., 2009; Joshi et al., 2015, 2016, 2020, 2021; Jiang et al., 2021). T3SS is one of the key virulence factors in many Gram-negative bacteria including *Dickeya*. Since it is well conserved, T3SS could serve as a good candidate target for the development of novel antibacterial agents (Baron, 2010; Kline et al., 2012). In this work, five phytohormone and seven chemical compounds were evaluated for their suppression of T3SS gene expression in *D. zeae* MS2. Among these, five plant natural products SA, PHBA, CA, PCA, and HA suppressed T3SS gene expression, and we also demonstrated that these compounds are able to suppress the HR of MS2 on non-host tobacco leaves and disease symptoms on host crops. It would be important for agricultural production to have a deeper understanding of how they affect the function of the T3SS in *Dickeya*.

As a master regulator of T3SS, HrpL is transcriptionally regulated by the HrpX/HrpY-HrpS-RpoN pathway and post-transcriptionally regulated by the GacS/GacA-RsmB-RsmA pathway (Yuan et al., 2020). In which, RpoN, a σ^54^-containing RNA polymerase holoenzyme, interacts with the σ^54^ enhancer-binding protein HrpS and initiates the transcription of *hrpL* (Chatterjee et al., 2002; Yap et al., 2005; Figure 5B).

We further analyzed which specific pathway(s) is affected by the five screened compounds in *D. zeae* MS2. Results indicated that all the five compounds SA (0.15 mM), PHBA (0.4 mM), CA (0.2 mM), PCA (0.2 mM), and HA (0.5 mM) significantly inhibited the expression of the master regulator HrpL, and SA had the most obvious inhibitory effect on the expression of all of the tested T3SS genes (Figure 4). Besides transcriptional inhibition through the HrpX/HrpY-HrpS-RpoN pathway, it inactivated HrpL through the GacS/GacA-RsmB-RsmA pathway (Figure 5). As a signal molecule in plants, SA is required for the induction of systemic acquired resistance as well as the activation of defense responses against biotrophic and hemi-biotrophic pathogens (Bari and Jones, 2009). A number of studies have found that SA can change the motility, biofilm formation, exoenzyme activity, and the pathogenicity of bacteria (Joshi et al., 2015; Cattò et al., 2017; Ahmed et al., 2019). Furthermore, SA has also been reported to reduce the expression of *vir* genes and reset virulence *via* SghR/SghA pathway in *Agrobacterium tumefaciens* (Yuan et al., 2007; Anand et al., 2008; Wang et al., 2019). It has also been shown to be a QS inhibitor for multiple bacterial species, including *P. aeruginosa*, *Pectobacterium*, and *A. tumefaciens* (Chang et al., 2014; Subramoni et al., 2014; Joshi et al., 2016, 2020; Ahmed et al., 2019). Previous studies have found that SA affects the *hrpA* promoter activity of several bacterial pathogens, including *E. amylovora* and *P. aeruginosa* (Yamazaki et al., 2012; Khokhani et al., 2013). This study identified the specific pathways of SA affecting *hrpA* expression and thus the virulence of *Dickeya* spp. on crops.

Naturally occurring PHBA is produced mainly by plants. Early studies have shown that PHBA has antibacterial and antioxidant activities against a variety of Gram-positive and Gram-negative bacteria (Rice-Evans et al., 1996; Cho et al., 1998). Recently, it was found that PHBA significantly downregulated the transcription of some genes in *hrp*/*hrc* gene cluster of *P. syringae* pv. *tomato* DC3000, and alleviated the disease symptoms of tomato leaves (Kang et al., 2020). In this study, PHBA did not affect the growth of *D. zeae* MS2 or alter the *hrpX*, *hrpY*, and *hrpS* mRNA levels, but obviously reduced the mRNA levels of *rpoN*, *gacS*, *gacA*, and other downstream regulatory genes of *hrpL* (Figures 4, 5A), suggesting that PHBA may directly act on *gacS* to inhibit the transcription of *hrpL*.

Cinnamyl alcohol and PCA, like PHBA, did not play a role through the two-component signal transduction system HrpX/HrpY, but inhibited the expression of *hrpS*, *rpoN*, and *gacS* (CA did not affect *gacS*), indicating CA and PCA inhibit *hrpL* transcription through both *hrpS* and *gacA*. PCA has been shown to inhibit the transcription of *D. dadantii* T3SS through HrpX/HrpY-HrpS-HrpL regulatory pathway (Li et al., 2009), rather than through the global regulator GacS/GacA, but our data revealed significantly lower *gacS* and *gacA* mRNA levels in cells grown in LS5 medium supplemented with PCA in comparison with DMSO control (Figure 5A). The contradiction may be attributed to the different signal transduction pathways of different strains. These results suggest that PCA inhibits *hrpL* through both the HrpS-RpoN (but not HrpX/HrpY) and the GacS/GacA pathways, and consequently lowers the expression of HrpL regulon genes. CA has also been shown to affect the *hrpA* promoter activity of *D. dadantii* and *E. amylovora*, but whether they alter other T3SS gene expression has not been confirmed (Khokhani et al., 2013; Li et al., 2015).

Hydrocinnamic acid has been previously discovered to affect the *hrpA* promoter activity of *E. amylovora* (Khokhani et al., 2013). In *P. aeruginosa*, HA inhibited QS and its regulatory genes (Sharma et al., 2019). In this study, HA showed a similar regulatory pattern to PCA except the unobvious effect on the expression of *hrpN* and *hrcC*, and a weak effect on *hrpY* (Figure 4).

In *P. syringae* pv. *tomato* DC3000, *gacA* mutation significantly attenuated the transcription of *rpoN*, but regulatory mechanism was unknown (Chatterjee et al., 2003). Another study found that the expression of the *gacA* global regulatory gene was significantly increased during the entire growth cycle in a *rpoN* mutant of *P. aeruginosa* PAO1 (Heurlier et al., 2003). This indicates that *gacA* and *rpoN* are correlated in some bacteria. Our study revealed the coordinate regulation patterns of *gacA* and *rpoN* (Figure 5A), suggesting the correlation of them in regulating T3SS in *D. zeae* MS2. In the ΔgacA mutant, *rpoN* expression decreased by 1.96-fold, suggesting positive regulation of GacA on the transcription of *rpoN* (Supplementary Figure S4), similar to that in *Erwinia* spp., *Pantoea stewartii*, and *P. syringae* (Chatterjee et al., 2003; Tang et al., 2006). Whether RpoN regulates *gacA* needs further study.

Type III secretion system is typically characterized by direct injection of T3SEs into host cells, leading to disease resistance of host plants and HR reaction. In our previous study, 50 putative T3SEs were predicted in MS2 genome based on a combination of four state-of-the-art bioinformatic tools (i.e., Bastion3, BEAN2, DeepT3, and pEffect), and 13 of which was demonstrated to be positively regulated by HrpL (Hu et al., 2022). Among them, only six include *hrp* box (GGAACC/T-N15/16-C/T/GCACNNA) in their promoter regions, such as DspE, HrpN, HrpW, HrpZ, HrpK, and HrpJ (Hu et al., 2022). In *Dickeya* bacteria, only DspE, HrpN, and HrpW have been characterized to be secreted *via* T3SS (Glasner et al., 2011). Although, we did not test the secretion of these T3SEs, it is probably affected under the treatment of the five inhibitors, since the expression of *dspE*, *hrpZ*, *hrpK*, and *hrpN*, except *hrpN* by HA treatment, was significantly reduced (Figure 4). Furthermore, from our recent study, T3SEs also function as virulence factors promoting the development of tissue maceration on host plants (Hu et al., 2022). HR is a programmed cell death reaction. After syringe infiltration in the tobacco, we found that MS2 strain treated with the five compounds showed weaker HR compared to the control (Figure 3). Specifically, the inhibition rate of SA, PCA, and HA on HR at least 94% (Figure 3), consistent with the result that most T3SS gene expression is inhibited (Figure 4). PHBA and CA also suppressed the HR remarkably. Finally, virulence assays *in planta* were performed to evaluate the inhibitory effects of five inhibitors in suppressing soft rot symptoms caused by different *Dickeya* pathogens on host plants. According to our results, the five compounds showed particularly obvious inhibitory effects on virulence in different species of *Dickeya* bacteria (*D. dadantii* 3,937, *D. fangzhongdai* CL3, *D. oryzae* EC1, and *D. zeae* MS2; Figure 6). It has been previously reported that cinnamic acid and SA affect the QS machinery of *Pectobacterium*, and completely suppress disease symptoms (Joshi et al., 2016). In our study, five plant-derived compounds failed to completely suppressed soft rot symptoms. This might be because other virulence factors or regulatory factors were still functional.

It is an important way to develop biosafety control agents from plant or microbial origin that inhibit plant pathogenic bacteria T3SS. In our study, we have shown the inhibitory effects of five plant natural products SA, PHBA, CA, PCA, and HA on the T3SS of MS2 both *in vitro and in planta*. They significantly inhibited the *hrpA* promoter activity and reduced the expression level of T3SS genes without affecting the growth and survival of *D. zeae* MS2. Furthermore, these compounds have been proven to be effective in attenuating the soft rot symptoms caused by different species of *Dickeya* bacteria on host crops. We also elucidated their possible regulatory pathways. The regulatory pathways of all of the compounds identified that were active against *D. zeae* have never been identified previously. This study also reports the universal functions of plant natural products SA, PHBA, CA, PCA, and HA on reducing the virulence of soft rot *Dickeya* bacteria. These results indicate that they play an important role in host–microbe interaction and have the potential to be used as natural, safe, and effective plant-derived biocontrol agents to cure plant diseases caused by *Dickeya* pathogens.

## Data Availability Statement

The original contributions presented in the study are included in the article/Supplementary Material, further inquiries can be directed to the corresponding author.

## Author Contributions

JZ conceived and designed the experiments. MH constructed the reporter plasmid MS2(pPhrpA-GFP). AH and XT screened the compounds and performed RNA extraction, cDNA synthesis, and qRT-PCR analysis. AH, MH, SC, and YX performed the pathogenicity tests and analyzed the results. AH, JZ, and MH wrote and revised the manuscript. All authors contributed to the article and approved the submitted version.

## Funding

The work was financially supported by grants from the Key-Area Research and Development Program of Guangdong Province (2020B0202090001 and 2018B020205003), the Guangzhou Basic Research Program (202102080613), the National Natural Science Foundation of China (31972230), the Natural Science Foundation of Guangdong Province, China (2020A1515011534), the Science and Technology Planning Project of Shaoguan City (200805094530618), and the Cultivation of Guangdong College Students’ Scientific and Technological Innovation (pdjh2019b0082).

## Conflict of Interest

The authors declare that the research was conducted in the absence of any commercial or financial relationships that could be construed as a potential conflict of interest.

## Publisher’s Note

All claims expressed in this article are solely those of the authors and do not necessarily represent those of their affiliated organizations, or those of the publisher, the editors and the reviewers. Any product that may be evaluated in this article, or claim that may be made by its manufacturer, is not guaranteed or endorsed by the publisher.
