# Supplementary material for: Five Plant Natural Products Are Potential Type III Secretion System Inhibitors to Effectively Control Soft-Rot Disease Caused by Dickeya
Source: Front Microbiol. 2022 Feb 22;13:839025. doi: 10.3389/fmicb.2022.839025 (PMC8901885; doi:10.3389/fmicb.2022.839025)
Supplement: Supplementary file 1 [file Data_Sheet_1.doc]

**Supplemental Material**

**Five plant natural products are potential T3SS inhibitors to effectively control soft-rot disease caused by *Dickeya***

Anqun Hu^1^, Ming Hu^1^, Shanshan Chen^1^, Yang Xue ^1^, Tan Xu^1^, Jianuan Zhou^1,*^

**^1^**Guangdong Province Key Laboratory of Microbial Signals and Disease Control, Integrative Microbiology Research Center, South China Agricultural University, Guangzhou 510642, China.

* Corresponding authors: jianuanzhou@scau.edu.cn

**Contents:**

**Supplementary Table S1**

**Supplementary Fig. S1**

**Supplementary Fig. S2**

**Supplementary Fig. S3**

**Supplementary Fig. S4**

**Table S1.** The strains, plasmids, and DNA primers used in this study

| **Objective** | **Characters or sequences (5’-3’)** | **Source or reference** |
| --- | --- | --- |
| **Strains** |  |  |
| ***Dickeya zeae*** |  |  |
| MS2 | Wild-type strain isolated from banana | Hu et al., 2018 |
| MS2(pPhrpA-gfp) | MS2 containing p*hrpA*, Km^r^ | This study |
| ***D. oryzae* EC1** | Wild-type strain isolated from rice | Zhou et al., 2011 |
| ***D. dadantii* 3937** | Wild-type strain isolated from *Saintpaulia ionantha* | Kotoujansky et al., 1982 |
| ***D. fangzhongdai* CL3** | Wild-type strain isolated from taro |  |
| ***Escherichia coli*** |  |  |
| DH5α | *supE44ΔlacU169* (ϕ80*lacZΔM15*), *hsdR17 recA1 endA1 gyrA96 thi-1 relA1* λ*pir* | Laboratory collection |
| CC118 | *Δ(ara-leu) araD ΔlacX74 galE galK phoA20 thi-1 rpsE rpoB argE (Am) recA λpir* | Laboratory collection |
| HB101(pRK2013) | *Thr leu thi recA hsdR hsdM pro*, Km^r^ | Laboratory collection |
| **Plasmids** |  |  |
| pPROBE-NT | Promoter-probe vector, Km^r^ | Laboratory collection |
| phrpA | pProbe-NT derivative with PCR fragment containing 490 bp hrpA promoter region, Km^r^ | This study |
| pKNG101 | R6K ori; *sacB* (Suc^s^), Sm^r^ | Kaniga et al., 1991 |
| **Primers** |  |  |
| ***Gene deletion*** |  |  |
| gacA-1 | ctgcaggtcgacggatccTATTATACCATACATGAC | This study |
| gacA-2 | CAGTCAGTCACTACTCAAAGAACAATTCTCC | This study |
| gacA-3 | GGAGAATTGTTCTTTGAGTAGTGACTGACTG | This study |
| gacA-4 | actatagactatactagtTCGCCACGAGGTCGGGTT | This study |
| gacA-F | TCAGCATCAACGTCGTCAGT | This study |
| gacA-R | TTCCAAGCACAGAAGCCAATG | This study |
| ***RT-PCR*** |  |  |
| atpD-F | TACCACGAAATGACCGACTCC | This study |
| atpD-R | CATTTCTTCTGCCAGTGTCG | This study |
| dspE-F | AGAAATGCAGTCGCTGACCA | This study |
| dspE-R | GGCTATCCTGGTTCTGACCG | This study |
| hrpZ-F | CAGAAAGGTCAGTTCGGCCA | This study |
| hrpZ-R | AGCTCGCTGGCGTTATTACT | This study |
| hrpK-F | TTACCAGCGGCATCCATTGT | This study |
| hrpK-R | AAACAAAAGGGCGTGCTGTC | This study |
| hrpN-F | GCGAAAGCGCTGAGTAAACC | This study |
| hrpN-R | TATCTTGTCACCGACGACGC | This study |
| hrcC-F | GAGCGGGTGGCGAATGTA | This study |
| hrcC-R | TTCCTCGACATGAAACCCACC | This study |
| hrpA-F | ACTGTCTAACGCTGCTGCTA | This study |
| hrpA-R | CAGAACTGGATGGCTTTGGC | This study |
| hrpS-F | CATCAACTTTCCGGTTGCCC | This study |
| hrpS-R | GTTTGGGTGGCGACAATCAC | This study |
| hrpY-F | CGCGCTATATTGACCACAGC | This study |
| hrpY-R | TCAGACGATTAAACGCTGGCT | This study |
| hrpX-F | GAGTGGCTGCGTGATGATTTT | This study |
| hrpX-R | AATCATGCCGTCGCGATTTT | This study |
| hrpL-F | GCACTTGCCGGAGGACA | This study |
| hrpL-R | GGAAAGACGCGAACGAACAG | This study |
| hrcV-F | CACAATCCGGCGTTGATCTG | This study |
| hrcV-R | CAGTTCAAACAGGCGCTCAA | This study |
| rpoN-F | TGATGTGGCAGGTGGAGTTG | This study |
| rpoN-R | CGATGCTCTCACGGATGTCA | This study |
| rsmA -F | TGGTCGTCTGAGTGTGATGG | This study |
| rsmA-R | AGGCTTCCGTGGTAATCCG | This study |
| gacS-F | TGAGTGGAGAGCGTGAAGTG | This study |
| gacS-R | CGAGTGACAATGGCGATGGT | This study |
| gacA-F | GGCAGAAGGTGACGGAGAT | This study |
| gacA-R | CCATGACGAATCGCCAGATG | This study |


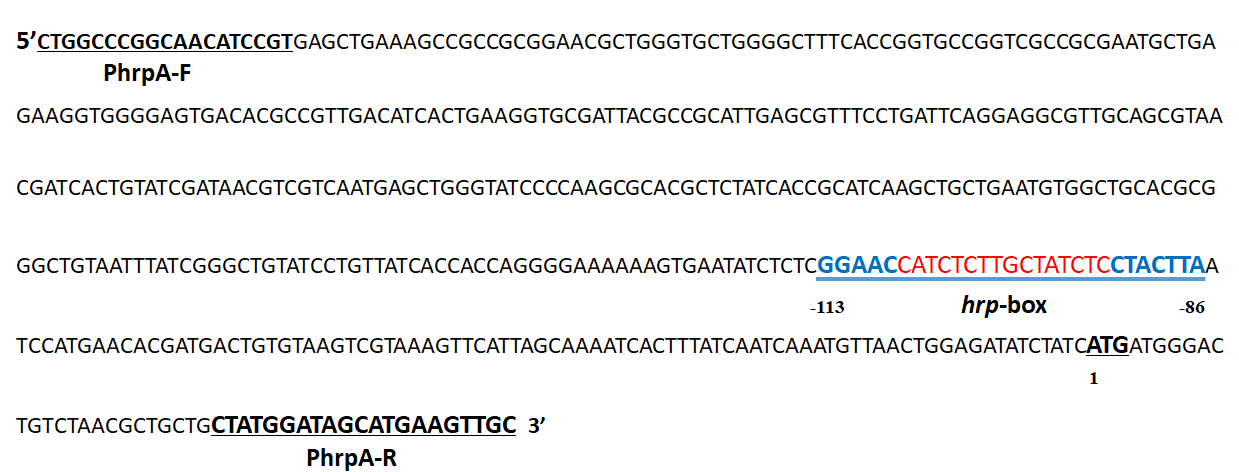


**Fig. S1** The promoter sequence of *hrpA* gene in *Dickeya zeae* MS2

**Fig. S2** The fluorescence intensity of LS5 medium supplemented with the compounds at the concentration of 0.2 mM.

**Fig.** **S3** Effects of SA (A), PHBA (B), CA (C), PCA (D) and HA (E) in different concentrations on the growth of MS2(pPhrpA-gfp) at 14 h. MS2(pPhrpA-gfp) culture (OD_600_ of 2.0) was transferred into the LS5 medium supplemented with different concentrations of tested compounds or DMSO at a ratio of 1:10, and then dispended into a 96-well tissue culture plate (100 μL per well) for cultivation in the dark for 14 h. OD_600_ was measured by a Multifunctional Microplate Reader. Three replicates were used in each compound and three independent experiments were performed with similar results. For statistical analysis, GraphPad Prism 8.4.3 software was used to perform Student’s *t*-test. Asterisks indicate statistically significant differences (ns, no statistical significance, * p < 0.05, ** p < 0.01).

**Fig. S4** Gene expression of *gacA* and *rpoN* in wild type MS2 and ΔgacA mutant. RNA was collected at a bacterial concentration of OD_600_ of 0.8 in LB medium. The cDNA levels of different samples were quantified by real-time PCR using a SYBR Green Master Mix. A housekeeping gene *atpD* was used as an endogenous control for data analysis. Then Student’s *t*-test analysis (Graphpad Prism 8.4.3) was performed on the gene expression. Three independent tests were performed with similar results (**** p < 0.0001).

Kaniga, K., Delor, I. and Cornelis, G. R. (1991) A wide-host-range suicide vector for improving reverse genetics in gram-negative bacteria: inactivation of the blaA gene of Yersinia enterocolitica. *Gene*, 109, 137-141.

Kotoujansky, A., Lemattre, M. and Boistard, P. (1982) Utilization of a thermosensitive episome bearing transposon TN10 to isolate Hfr donor strains of Erwinia carotovora subsp. chrysanthemi. *J Bacteriol*, 150, 122-131.
